# Comparative evaluation of anesthetic efficacy of warm, buffered and conventional 2% lignocaine for the success of inferior alveolar nerve block (IANB) in mandibular primary molars: A randomized controlled clinical trial

**DOI:** 10.15171/joddd.2018.016

**Published:** 2018-06-20

**Authors:** Reenu Sarah Kurien, Mousumi Goswami, Sanjay singh

**Affiliations:** ^1^Department of Pedodontics and Preventive Dentistry, I.T.S Dental College, Hospital and Research Center, Greater Noida, Uttar Pradesh, India; ^2^Department of Oral and Maxillofacial Surgery, Faculty of Dentistry, Jamia Millia Islamia, New Delhi, India

**Keywords:** Warm, buffered, IANB, pulp therapy, children

## Abstract

***Background:*** Maintaining primary teeth in the oral cavity is of prime importance, and grossly carious teeth may require pulp therapy for the same. Pain on injection and incomplete anesthesia causes failure of the procedure and result in fear and anxiety. Various methods have evolved to overcome this; such as distraction, topical anesthesia, etc. A few techniques regaining popularity in dentistry in recent times is the warming or buffering of the solution prior to administration. This study thus aimed to compare and evaluate the anesthetic efficacy and the patient’s pain reaction to pre-warmed, buffered and conventional 2% lignocaine for the success of Inferior Alveolar Nerve Block in mandibular primary molars undergoing pulp therapy.

***Methods:*** The study is a randomized, split-mouth trial. Sixty children between six -12 years, requiring pulp therapy bilaterally on mandibular primary molars, were administered conventional, buffered or pre-warmed 2% lignocaine on two separate appointments. Various parameters were assessed using objective and subjective scales.

***Results:*** Pre-warmed and buffered anesthetics had lesser pain on injection (p<0.001, p<0.001) and pulp therapy (p=0.001, p=0.014), faster onset of action (p=0.004, p=0.001), lower SEM Sound (p=0.035, p=0.028), Eye (p< 0.001, p=0.013) and Motor (p=0.008, p=0.021) scores and shorter duration of action (p< 0.001, p=0.015). No significant difference was found between the two modified solutions. Thus pre-warmed and buffered anesthetic solutions fared better than the conventional solution for all parameters but had no advantage over each other.

***Conclusion:*** Buffering or pre-warming the anesthetic solution reduces pain on administration and during procedures in children.

Trial registration number: CTRI/2017/02/007922

## Introduction

he management of pain is a crucial factor in pediatric dentistry since it dictates the behavior of the patient during the present appointment, as well as ensures compliance for future visits. The administration of local anesthesia (LA) is a prerequisite for reducing pain during various restorative, endodontic and minor surgical procedures and is more necessary in children than in adults. However, the very means employed to reduce pain in itself might be a source of both pain and anxiety in children. Increased anxiety can increase pain perception in children and may create a barrier to receiving optimal and necessary dental care. Various factors such as the speed of injection, pressure during administration, injection site, pH, volume and temperature of the anesthetic solution have been attributed to the degree of pain associated with local anesthetic administration.^1-5^

Various techniques have been suggested to alleviate pain during injection. These could range from behavioral techniques such as reframing, using distractions or providing positive suggestions. Other techniques sought to reduce pain via instrumental approaches such as topical anesthetic agents prior to the injection, placing lignocaine patches on the gingiva, using electronic dental anesthesia or computerized devices such as the Wand.^3^ Methods using instruments such as the vibraject^6^ and intraligamentaryinjections^7^ have been helpful adjuncts in reducing pain. Operator-defined techniques such as troncular injections, injection administration speeds below 1 tube per minute, and the compression of the tissue surrounding the puncture site have also been used.^8^ Warming the local anesthetic solution to body temperature^9^ has been found to effectively reduce pain during injection for eye surgery^10^ and plastic surgery and thus may have relevance in dentistry.^11^ Another technique that has been used in the past, but is fast regaining popularity in recent years, is to add a buffer to raise the pH of the anesthetic solution.

There is still ambiguity in the use of these techniques in the dental literature. Very few studies have been conducted on children using buffered solutions in dentistry, and only one study^3^ has been conducted on the use of warm solutions in children, using a topical anesthetic agent before administration of anesthesia to decrease pain.

This study was thus undertaken to compare the anesthetic efficacy of buffered and conventional 2% lignocaine on the success of IANB technique for primary molars requiring pulpal therapy in children aged 6‒12 years.

## Methods

This randomized, double-blind split-mouth study was conducted in the Department of Pedodontics and Preventive Dentistry. Approval from the Institutional Ethics Committee was obtained before conducting the research under the code IEC/Pedo/13/15; the trial registration code was CTRI/2017/02/007922. The study was undertaken in full accordance with ethical principles, including the World Medical Association Declaration of Helsinki.

Sample size was estimated by nMaster software (version 2, CMC Vellore) and was estimated using the formula

N = Z^2^[2 S_P_^2^] / d^2^

where, Z is the standard normal deviate for alpha error of 5% or 0.05 = 1.96, S_P_^2^ = Pooled standard deviation = S_1_^2^+ S_2_^2^ / 2, S_1_ = SD in group 1 (warm LA) = 2, S_2_ = SD in group 2 (LA at room temperature) = 2, d = Difference in mean (onset of anesthesia in minutes) between two groups = 1.2

Using this formula, a sample size of forty-two 6‒12-year-olds was decided upon. Considering possible drop-outs, it was increased to 60 participants, i.e. 30 in each group. The participants were selected from the outpatient department, who were indicated for at least two clinical sessions of pulp therapy procedures (pulpectomy or pulpotomy) requiring IANB anesthesia. Only children who exhibited Frankel’s behavior rating grade of 3‒4 and did not require any sedation were selected. Children with a history of medically compromising conditions, allergy to lignocaine, or any abscess or swelling at the site of the tooth involved were excluded from the study. Written informed consent was obtained from the parents before enrolling the children in the study and only those agreeing to comply with the treatment visits and protocol were included.

On the first visit, the children were subjected to a thorough check-up, and radiographs of the offending teeth were taken. Children who had mandibular primary molars of both right and left sides affected with deep dental caries, requiring pulp therapy, were selected for the study. They were also shown the Wong Baker Faces Pain Scale (WBFPS) and familiarized with it so as to effectively enable them to rate their pain perception using it. When pulp therapy was initiated it was noted whether there was any bleeding from the pulp or not. If the pulp did not show bleeding (indicative of nonvital pulp), the tooth was excluded from the study.

The study was carried out with the help of two investigators. The first investigator randomized the participants in accordance with the CONSORT 2010 guidelines^12^ by asking them to choose one of two differently colored balls. This would determine which modified preparation of local anesthetic solution would be administered (either warm or buffered). The participants were then asked to choose from two other differently colored balls to determine which solution would be administered on the first appointment (conventional or the previously chosen modified solution). The alternate preparation was administered after a gap of one week on the contralateral side to negate any carry-over effect^13,14^

While administering the IANB, 1.8 mL of the solution was administered over 60 seconds, as recommended by Malamed^15^ using a 27-gauge needle.

In order to make a buffered solution, a 30-mL vial of 2% lignocaine anesthetic solution with 1: 200,000 epinephrine (Lox two percent, Neon Laboratories Ltd., Mumbai, India) was taken and from it 3 mL of solution were removed using a standard disposable syringe. To this, 3 mL of 8.4% sodium bicarbonate were added, using a sterile, standard disposable syringe to achieve a dilution of 1:10. It was shaken until the solution was clear, to ensure that sodium bicarbonate dissolved completely.

To pre-warm the solution, the methods described by Allen et al^16^ and Davidson et al^17^ were combined. Five mL of commercially available 2% lignocaine hydrochloride with 1:200,000 epinephrine (Lox two percent, Neon Laboratories Ltd., Mumbai, India) were placed in a 5-mL vial and warmed in a thermostatically controlled water bath up to 41°C; then 2 mL of the anesthetic solution were loaded into a 3-mL syringe and administered to the patient (within 30‒40 s) after it attained body temperature (37°C). Another vial with the same anesthetic solution warmed to the same temperature was also prepared. A thermometer was used to check the temperature of this second vial to ascertain the temperature that the solution had attained just before the contents of the first vial were administered for the nerve block.

The second investigator, who was blinded, assessed the pain on administration of anesthesia by asking the patients to rate their pain reaction on the WBFP scale just after the block was rendered and also carried out gingival probing every 15 seconds to assess the time of onset of anesthesia. After rubber dam isolation, access cavity was prepared and pulp therapy was initiated by the first investigator. At this point, patients were again asked to rate their pain reaction on the WBFP scale by the second investigator, who also observed the patients’ reactions during pulp therapy, using the Sound, Eye, Motor (SEM) scale. This investigator had been calibrated for the SEM scale prior to the commencement of the study, by evaluating the Sound, Eye and Motor scores of 10 patients, who were not included in the main study, while they received an IANB.

The pulp therapy was completed as indicated and the teeth were restored using glass-ionomer cement and at a later date, using a stainless steel crown.

The children were asked to stay in the department for three hours, to assess the duration of anesthesia. If the anesthesia did not worn off by the third hour, telephone conversations with the parents were held after the fifth hour of completion of the procedure to evaluate the duration of anesthesia.

On the second visit, the other anesthetic solution was administered on the contralateral side and evaluated for the same parameters.

Three children did not return for the follow-up appointment in the buffered anesthesia group, one did not return in the pre-warmed group and one tooth showed no bleeding from the pulp during access cavity preparation. These children were excluded from the study, leaving 27 and 28 children in both groups respectively, leading to a final sample size of 55.

The data collected for all the parameters were then subjected to statistical analysis using the Wilcoxon signed-rank test and Mann-Whitney U tests and the results were then formulated.

## Results

Sixty 6‒12-year-old children participated in the study. However, as a result of drop-outs and exclusion due to nonvital pulp, the total sample size decreased to 55. Thus, a total of 110 IANB injections were administered.

### Pain on administration

**Figure 1:**
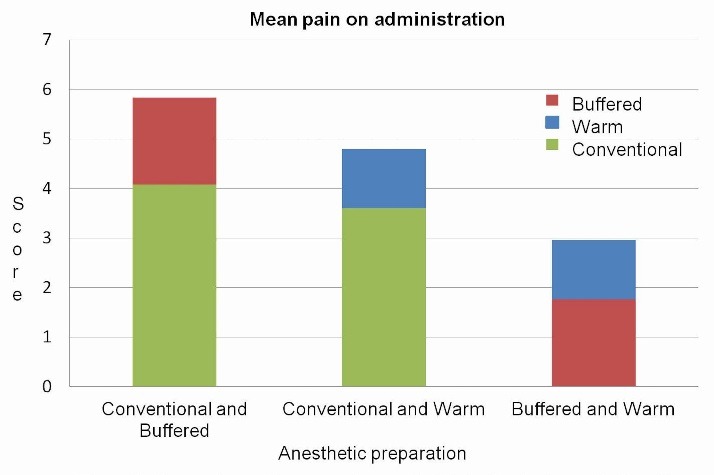
Comparison of mean pain scores on administration between buffered, conventional and warm preparations

Comparison of mean pain scores on administration was carried out between the conventional and modified solutions, revealing that both pre-warmed and buffered preparations resulted in significantly less pain on administration as compared to the conventional solution (P<0.001, P<0.001) (Figure 1).

### Onset of anesthesia

**Figure 2:**
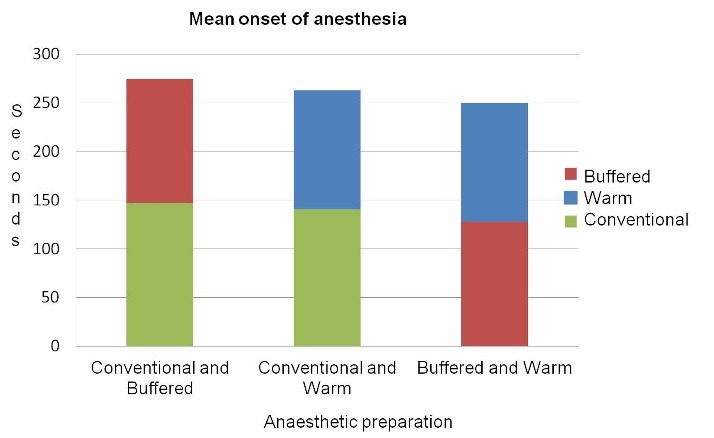
Comparison of mean onset times of anesthesia between buffered, conventional and warm preparations

Comparing of the mean times of the onset of anesthesia showed a faster onset of action for both pre-warmed and buffered solutions (P=0.004, P=0.001) (Figure 2).

### Pain on pulp therapy

**Figure 3:**
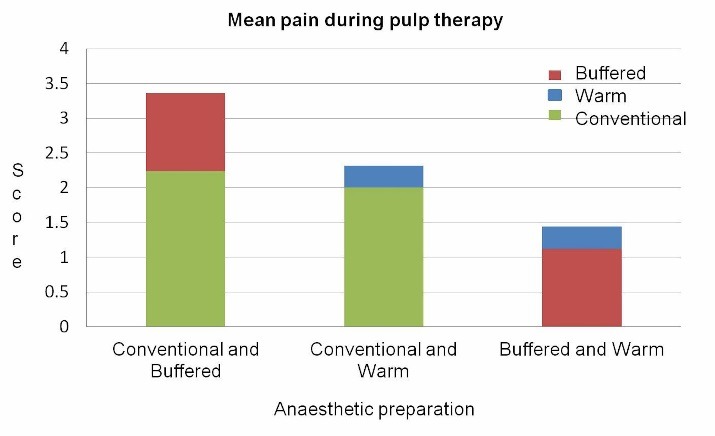
Comparison of mean pain scores during pulp therapy for buffered, conventional and warm preparations

Comparison of mean pain scores during pulp therapy procedure showed more pain with the use of the conventional anesthetic solution (P=0.001, P=0.014) (Figure 3).

### SEM score

Comparison of the mean sound, eye and motor scores using the SEM scale showed significant decreases in sound (P=0.035, P=0.028), eye (P< 0.001, P=0.013) and motor scores (P=0.008, P=0.021) with both modified preparations (Table 1-3).

**Table 1:** SEM scores for buffered and warm solutions

	**Conventional**		**Buffered**				
	**Mean**	**SD**	**Mean**	**SD**	**SD**	**Z-value**	**P-value**
**SEM Sound** **score**	0.72	0.79	0.32	0.75	0.40	-2.202	0.028*
**SEM Eye** **score**	0.72	0.74	0.36	0.57	0.36	-2.496	0.013*
**SEM Motor** **score**	0.68	0.75	0.36	0.49	0.32	-2.309	0.021*

**Table 2:** SEM scores for conventional and warm solutions

	**Conventional**		**Warm**				
	**Mean**	**SD**	**Mean**	**SD**	**Mean difference**	**Z-value**	**P-value**
**SEM Sound** **score**	0.52	0.65	0.12	0.33	0.40	-2.111	0.035*
**SEM Eye** **score**	0.92	0.64	0.24	0.44	0.68	-3.690	<0.001*
**SEM Motor** **score**	0.64	0.81	0.16	0.37	0.48	-2.652	0.008*

**Table 3:** SEM scores for buffered and warm solutions

	**Buffered**		**Warm**				
	**Mean**	**SD**	**Mean**	**SD**	**Mean difference**	**Z-value**	**P-value**
**SEM Sound** **score**	0.32	0.75	0.12	0.33	0.20	-0.853	0.394
**SEM Eye** **score**	0.36	0.57	0.24	0.44	0.12	-0.696	0.486
**SEM Motor** **score**	0.36	0.49	0.016	0.37	0.20	-1.596	0.111

### Duration of anesthesia

**Figure 4:**
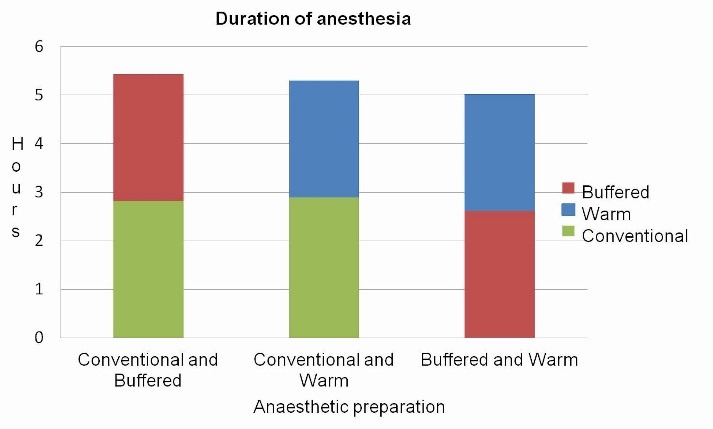
Comparison of duration of anesthesia between buffered, conventional and warm preparations

Comparison of the mean durations of anesthesia revealed significantly shorter duration (P<0.001, P=0.015) with the use of pre-warmed and buffered preparations (Figure 4).

However, when the two modified preparations were compared, no statistically significant difference was found between the two for any of the parameters considered.

## Discussion

Dental procedures induce various negative responses such as stress, anxiety and pain in children.^1^ Approximately 20% of children have been reported to have dental fears and 21% exhibit negative behaviors in the dental settings.^18^ Children undergoing dental procedures often display crying, screaming, groaning and verbalization of their anxiety and pain. In such stressful conditions, it is helpful to use preventive preparatory interventions, which might disrupt a cycle whereby the painful experience leads to negative memories, which can produce greater anxiety and pain response.^19^ Our study was thus an endeavour to find a possible solution to reduce pain and anxiety associated with the administration of local anesthesia using a conventional syringe and needle.

This study showed a significant difference in children’s objective assessment and the subjective reaction to the use of room-temperature or warmed local anesthetic solutions and to non-buffered and buffered solutions. Our study reported that there was a significant reduction in pain on administration and during pulp therapy on the WBFP scale and a faster onset and shorter duration of anesthesia for both modified preparations. The SEM scale criteria showed that the participants had better acceptance of buffered or warm anesthetic solutions as compared to un-modified solutions.

There are several drawbacks associated with the administration of local anesthetics. They are unreliable in areas of inflammation and infection, have a relatively slow onset of action and can cause post-injection tissue trauma. The most common complaint by patients in dentistry is the burning and stinging of injections due to the acidic anesthetic agents. This is because synthetic local anesthetics are prepared as weak bases and might precipitate as insoluble powdered unstable solids. Thus they are combined with an acid to form a salt that is water-soluble; these can be dissolved in sterile water or saline, creating a stable, injectable anesthetic solution.^20^

The body’s pH is maintained at 7.4. Injectable local anesthetics without vasoconstrictors have a slightly acidic pH of around 6.4 which is relatively close to the body’s physiologic pH Vasoconstrictors are added to anesthetics to improve both the depth and the duration of analgesia and also to allow for more prolonged pulpal analgesia. However, adrenaline oxidizes rapidly at or near physiologic pH, so an antioxidant (most commonly sodium bisulfite) is added to the solution, lowering the pH to approximately 3.5. Studies have reported that in general, the anesthetic pH ranges from 2.86 to 4.16. By comparison, lemon juice has a pH range of 2.2 to 2.6. Thus it is expected that bathing a needle wound in the patient's mouth with an anesthetic solution at pH=2.9 can create a significant amount of pain.^21^

The literatureon local anesthetics has reported the benefits of alkalinization for more than 100 years, and anesthetic buffering has been widely accepted in medicine as a way to make local anesthetic injections more comfortable. Adding sodium bicarbonate to the anesthetic solution aids in increasing its alkalinity and in eliminating the problems associated with an acidic solution. In our study, we added 8.4% sodium bicarbonate to the solution to increase its alkalinity.

The findings of our study were similar to studies conducted by Christoph et al^22^ and McKay et al,^23^ who found that buffered anesthetic solutions with sodium bicarbonate significantly decreased pain of injection. However, this was in contrast to studies by Gershon et al^24^ and Burns et al,^25^ who found no significant pain reduction with buffered anesthetic solutions for intradermal anesthesia. Cristoph et al^22^ and Whitcomb et al^26^ found no difference in onset time,though DiFazio et al^27^ andSinnott et al^28^ found that anesthetic formulations with higher pH values had a faster onset.

Studying its effects on pulpal anesthesia, Bowles et al^25^ found less pain with buffered lignocaine, but Primosch and Robinson,^29^ Whitcomb et al^26^ andChopra et al^1^ found no decrease in pain.

Considering the onset of anesthesia, the results of the present study were similar to those of a study by Brennan et al,who reported that an acidic solution produced a longer duration of anesthesia while the alkaline solution had the shortest duration (Matthew B. Balasco thesis)

Raising the temperature of the anesthetic solution prior to administration has also been shown to decrease the degree of pain perceived by patients. The reason for this is not known, but there are several possibilities. Nerve endings are sensitive to cold, and warming the injection might directly reduce stimulation; alternatively, the warmer injection might increase the rate of onset of the block and inhibit pain transmission before the nociceptive impulses are fully appreciated.^17^ Another explanation is that the pKa (dissociation constant) value of local anesthetics are temperature-dependent and when warmed, this pKa value decreases,^16^ allowing more of the active deionized form of the anesthetic to penetrate into the nerve membrane. This form of the local anesthetic agent produces rapid anesthesia and also helps reduce pain during injection. According to Powell,^30^ pKa of lignocaine is 7.57 at 40°C and 7.92 at 25°C. Thus warming of lignocaine might increase the speed of onset and the quality of local anesthesia.

Alonso et al^31^ reported an inverse relationship between temperature and pain and reported that the highest mean pain level occurred at 10°C followed by 18^o^C, 37°C and 42^o^C.

Our study had results similar to that conducted by Bainbridge et al,^11^ who compared the effect of local anesthetics at room temperature and body temperature for procedures on the cheek and chin and found that patients perceived significantly less pain when injected with local anesthetic agents at body temperature.

The results were also similar tothose reported by Rogers et al, who found in a study conducted with dental students aged 22 to 32 years, using the VAS measure, that the warmed dental injection was significantly more comfortable than the ambient-temperature injection (Ulu et al, Comparison of The Effect of Room Temperature and Warmed Local Anesthetic Solution for Dental Procedure- manuscript)

In accordance with our study,Westblade^32^ in 1968 suggested that the speed of onset and degree of anesthesia might decrease with cool solutions.

The findings were contrary to a study conducted by Ulu et al, who compared warmed anesthetic solutions and solutions at room temperature in a split-mouth design on 46 adult patients requiring extraction of the third molar and found no statistically significant difference between the two groups. (Ulu et al. Comparison of The Effect of Room Temperature and Warmed Local Anesthetic Solution For Dental Procedure- manuscript)

A study by Ram et al^3^ also showed contrary results while comparing warm and room-temperature anesthetics for dental procedures and found no significant differences between the two.A recent systematic review and meta-analysis by Hogan et al^33^ concluded that more research was required to have conclusive evidence on the benefits of warming anesthetic solutions in dental procedures and its effect on children.

We used the Wong Baker^34^ scale for subjective pain assessment in our study since it has been reported to be effective in children 3‒16 years of age and can be more easily understood than the visual analogue scale (VAS) scale.We also used thesound, eye and motor scale for a correct subjective evaluation of the pain.

Our study is one of the few studies conducted on children for dental treatment using buffered and pre-warmed anesthetic agents, without prior administration of any topical anesthetics, for pulp therapy procedures; the results showed that modified solutions were superior in all the parameters considered, both clinically and statistically

## Conclusion

The findings of our study led us to conclude that 2% lignocaine with 1:200,000 epinephrine warmed to 37°C or buffered with 8.4% sodium bicarbonate reduces pain on injection during IANB technique and pain on pulp therapy in children compared with the unmodified anesthetic solution and also provided significant clinical advantage in relation to the onset and duration of anesthesia for IANB in children. Thus modifying the anesthetics might be a useful clinical tool in not only making procedures requiring anesthesia more acceptable to the patient but also in improving their compliance during dental visits.

## Acknowledgements

We would like to thank and acknowledge Dr. Ruchi Nagpal and Dr. Sukhvinder Singh Oberoi for helping with the statistical analysis of the study.

## Author contributions

MG and RSK conceptualized the design of the study and carried out the clinical research. RSK wrote the manuscript and MG and SS settled and finalized the writing of the research paper.

## Funding

The study was self-funded.

## Competing interests

The authors declare no competing interests with regards to the authorship and/or publication of this article.
